# Transparent functional nanocomposite films based on octahedral metal clusters: synthesis by electrophoretic deposition process and characterization

**DOI:** 10.1098/rsos.181647

**Published:** 2019-03-06

**Authors:** Ngan Thi Kim Nguyen, Marion Dubernet, Yoshio Matsui, Maxence Wilmet, Naoto Shirahata, Gaulthier Rydzek, Noée Dumait, Maria Amela-Cortes, Adèle Renaud, Stéphane Cordier, Yann Molard, Fabien Grasset, Tetsuo Uchikoshi

**Affiliations:** 1 Research Center for Functional Materials, National Institute for Materials Science (NIMS), 1-2-1 Sengen, Tsukuba, Ibaraki 305-0047, Japan; 2 CNRS-Saint-Gobain-NIMS, UMI3629, Laboratory for Innovative Key Materials and Structures (LINK), National Institute for Materials Science (NIMS), 1-1 Namiki, Tsukuba 305-0044, Japan; 3 Research Center for Materials Nanoarchitectonics (MANA), National Institute for Materials Science (NIMS), 1-1 Namiki, Tsukuba 305-0044, Japan; 4 Univ Rennes, CNRS, ISCR – UMR 6226, 35000 Rennes, France; 5 Centre for Research on Adaptive Nanostructures and Nanodevices (CRANN) and Advanced Materials Bio-Engineering Research Centre (AMBER), School of Chemistry, Trinity College Dublin, Dublin, Ireland

**Keywords:** niobium cluster, molybdenum cluster, photoluminescence, NIR, electrophoretic deposition

## Abstract

Transparent optical thin films have recently attracted a growing interest for functional window applications. In this study, highly visible transparent nanocomposite films with ultraviolet (UV)-near-infrared (NIR)-blocking capabilities are reported. Such films, composed of Mo_6_ and Nb_6_ octahedral metal atom clusters (MC) and polymethylmethacrylate polymer (PMMA), were prepared by electrophoretic deposition on indium tin oxide-coated glass (ITO glass). PMMA was found to improve both the chemical and physical stability of Mo_6_ and Nb_6_ MCs, resulting in a relatively homogeneous distribution of the clusters within the PMMA matrix, as seen by microstructural observations. The optical absorption spectrum of these transparent MC@polymer nanocomposite films was marked by contributions from their Mo_6_ and Nb_6_-based clusters (absorption in the UV range) and from the ITO layer on silica glass (absorption in the NIR range). Mo_6_@PMMA nanocomposite films also exhibited excellent photoluminescence properties, which were preserved even after exposure to 50°C at a relative humidity of 70% for one month. These films cumulate high transparency in the visible range with remarkable UV-NIR blocking properties and represent interesting candidates for functional glass application.

## 1. Introduction

Robust low cost and highly transparent functional nanocomposite thin films have recently attracted a focused attention owing to their potential for energy saving [1,2]. Prominent applications arising from such films include light and colour management devices [3], photovoltaic luminescent solar concentrators (LSC) [4–8] and window solar control [9–11]. For instance, LSC were extensively studied as electrical power generators for building-integrated photovoltaics [4–8] accompanying the development of ultraviolet (UV) and near-infrared (NIR) barriers for functional windows [2,4].

With this respect, a new generation of nanocomposite thin films has been recently investigated, including (i) red luminescent octahedral metal atom clusters (MC) with large Stokes shift and (ii) strong UV-NIR absorbers MC [3,4,12,13]. The term ‘metal atom cluster’ was introduced by F. A. Cotton in 1964 to define a finite group of metal atoms (two or more) that are held together by metal–metal bonds, in addition to being bonded to other non-metal ligands [14]. In the past decades, octahedral MC compounds have demonstrated a rich complexity of structural and physico-chemical properties. Thus, they have shown promising optical properties for energy conversion (molecule-like energy gaps, strong photoluminescence (PL) in NIR region, etc.), and electronic/electrochemical properties with strong potential for energy storage and supply (superconductivity, battery, thermoelectricity, hydrogen affinity, etc.) [15,16]. Typically, octahedral MC are composed of ${\left\{M_{6}L_{8}^{i}\right\}}^{4+}$ (M = Mo, W, Re) or ${\left\{M_{6}L_{12}^{i}\right\}}^{2+}$ (M = Nb, Ta) metallic cores (L^i^ = inner ligand), coordinated with halogen or chalcogen functional apical ligands (L^a^) [14–18]. In the reduced form, valence electron counts (VECs) are equal to 24 electrons or 16 electrons per ${\left[M_{6}L_{8}^{i}L_{6}^{a}\right]}^{2-}$ or ${\left[M_{6}L_{12}^{i}L_{6}^{a}\right]}^{4-}$ cluster unit, respectively. A strong absorption in both the UV and visible light (Vis) ranges was reported for ${\left\{Mo_{6}L_{8}^{i}\right\}}^{4+}$ cluster cores coordinating with ligands (L = Cl, Br, I or OCOC_n_F_2n+1_), resulting in a prominent luminescent emission within the deep red/NIR region [15–24]. The PL mechanism of such MC is typically ascribed to a type A chromophore absorption followed by separation between the absorption and emission peaks [20]. As a result, expanding the Stokes shift for reducing the overlap between the cluster's absorption and emission peaks has emerged as a critical challenge for improving the electric conversion efficiency of LSCs [3,4]. The excitation state of some MC also allowed producing singlet oxygen (^1^O_2_) for photodynamic therapy applications [18]. In parallel, octahedral MC based on ${\left\{M_{6}L_{12}^{i}\right\}}^{2+}$ core, including $K_{4}[Nb_{6}{\mathrm{Cl}}_{12}^{i}{\left(\mathrm{Cl}\right)}_{6}^{a}]$ (KNC) and $K_{4}[Nb_{6}Br_{12}^{i}{\left(\mathrm{Br}\right)}_{6}^{a}]$ (KNB) compounds, are well known as strong redox agents and UV-absorbents [25]. Over the past several decades, hexanuclear tantalum bromides have attracted considerable attention, in particular, as a commercial tool for the phase determination of large biological assemblies by X-ray crystallography and as radiographic contrast agents [26–29]. The charge of cluster units based on ${\left\{M_{6}L_{8}^{i}\right\}}^{4+}$ and ${\left\{M_{6}L_{12}^{i}\right\}}^{n+}$ (*n* = 2, 3, 4) metallic cores could also be tuned, improving their processability. This was typically achieved by tuning the central cation oxidation state or by coordinating with specific apical ligands. The resulting MCs can be synthesized in large amounts, by using reproducible processes and exhibit a superior solubility in host matrix materials. Both the processability of MC and the retention of their optical properties were recently improved by dispersing the clusters in organic and inorganic matrices, including photosensitive SU8 resist polymer [30], polyurethane [31], polystyrene [32], polyvinylpyrrolidone (PVP) [12,33], silica nanoparticles [34–36] and β-cyclodextrin [37]. In that respect, polymethylmethacrylate (PMMA) emerged as one of the best polymeric dispersion matrices, owing to its good chemical and thermal stability, excellent biocompatibility and prominent transparency in the UV-NIR absorption range [38,39]. Luminescent Mo_6_@PMMA hybrid nanocomposites were prepared by *in situ* polymerization of vinyl acrylate containing metal cluster compounds [40–43] or by using supramolecular interactions between a ternary $Cs_{2}[Mo_{6}I_{8}^{i}{\left(\mathrm{OCO}C_{2}F_{5}\right)}_{6}^{a}]$ (CMIF) clusters salt and PMMA partially functionalized by lateral poly(ethyleneoxide) chains [44]. Within such hybrid coating materials, the polymer matrix acted as a waveguide enabling both trapping the incident solar light and directing the light emitted by MC to its edges. This mechanism allowed reaching the best LSC performances for PV cells [4]. Among the various strategies available to disperse MC in polymer matrices (functionalization of the MC for covalent bonding, electrostatic interaction, direct homogeneous mixing, etc.), direct mixing is efficient and preserves reasonably well the intrinsic properties of the MC. Dispersing MC in polymer matrices enabled using polymer-processing technologies to shape the nanocomposites, including electrospinning [43] and pellet moulding [45]. When such nanocomposites were used as transparent thin films, they exhibited a large effectiveness for optical devices [5–7,12,39,46,47]. Several wet chemistry film deposition strategies were thus developed [48], including polymer-assisted deposition [49], sol-gel dip coating [50,51], spin coating [52], printing [44,53] and electrophoretic deposition (EPD) [54,55]. EPD quickly emerged as one of the best industry-friendly strategies owing to its ability to assemble homogeneous films at room temperature, within a short time and with a controlled thickness. Such advantages allowed preserving the original octahedral structure of the clusters and EPD, thus successfully enabled preparing pure and nanocomposite MC thin films respectively composed of highly optically effective Mo_6_ [56–59] and Ta_6_@PVP [13,59].

In this study, three types of highly transparent MC/PMMA nanocomposite thin films were prepared on indium tin oxide-coated glass (ITO glass) by EPD. Their optical properties were studied for potential window applications. CMIF clusters were used, owing to their excellent luminescence with high emission yields and long emission lifetimes in CH_3_CN at 298 K [60,61]. Octahedral clusters of KNC and KNB were used for their good UV-NIR absorbance, oxidizing and magnetic properties [62,63]. Surprisingly, no film deposition was possible with pure ${\left[Mo_{6}I_{8}^{i}{\left(\mathrm{OCO}C_{2}F_{5}\right)}_{6}^{a}\right]}^{2-}$, ${\left[Nb_{6}Cl_{12}^{i}{\left(\mathrm{Cl}\right)}_{6}^{a}\right]}^{4-}$ and ${\left[Nb_{6}Br_{12}^{i}{\left(\mathrm{Br}\right)}_{6}^{a}\right]}^{4-}$ clusters whereas EPD deposition was possible with [Mo_6_Br_14_]^2−^ and ${\left[Ta_{6}Br_{12}^{i}(H_{2}O)s_{6}\right]}^{2+}$ clusters [13,56–59]. Further investigations are in progress to understand this result. However, the use of PMMA as a dispersing medium greatly improved the film quality and stabilized the cluster's optical properties during the EPD process. This work reports on the successful preparation of 1.5 µm thick Nb_6_ and Mo_6_-based nanocomposite thin films with low polymeric loading. These coatings were homogeneous and exhibited good UV and NIR absorbing properties, cumulated with an improved stability against moderate temperature and humidity conditions.

## 2. Material and methods

### 2.1. Synthesis of clusters

$Cs_{2}{\left[Mo_{6}I_{8}^{i}{\left(\mathrm{OCO}C_{2}F_{5}\right)}_{6}^{a}\right]}^{}$ (CMIF) was prepared by following previously published results [31,64]. Hexanuclear niobium halide clusters ($K_{4}Nb_{6}X_{8}^{i}X_{6}^{a}$ with X = Cl or Br) were synthesized by solid-state chemistry at high temperature from the reaction of NbX_5_, NbX and Nb chemicals as proposed by Koknat *et al.* [65].

### 2.2. Preparation of the cluster@PMMA suspensions

Acetone was selected as a dispersing solvent for the nanocomposite suspension. PMMA (M = 350 000 g mol^−1^, Sigma-Aldrich Ltd) was dissolved in acetone (99.5%, Nacalai Tesque, Inc.) at a suitable concentration, and stirred with a magnetic stirrer for 24 h.

The CMIF cluster suspension was prepared in acetone at a concentration of 40 g per litre and stirred for 10 min. The PMMA (50 g per litre) and CMIF suspensions were prepared separately and mixed together with a 2 : 1 volume ratio, and agitated for 15 min leading to a transparent and homogeneous suspension. The wt% concentration of the CMIF in the last suspension containing PMMA used for the EPD is 28.6 wt%.

Nb_6_-based clusters, namely KNB and KNC, were dispersed at a concentration of 5 g per litre in methanol (99.5%, Nacalai Tesque, Inc.), acetone or an acetone/water (100 : 1) mixture, followed by ultrasonication for 1 h. Chemical impurities (estimated to 40% in KNC and KNB powders) were eliminated by 0.2 µm filtration, and the transparent yellow (in acetone), green (in methanol) and dark brown (acetone/water) suspensions were collected. The wt% concentration of Nb_6_ dispersed in the methanol and acetone suspensions is about 5.8 wt% after mixing with PMMA solution (100 g per litre) with a 2 : 1 volume ratio and continually stirred for 15 min. For the suspension prepared in acetone/water, before mixing with PMA solution, it was subsequently evaporated to increase the concentration of Nb_6_ clusters by 2 and mixed with PMMA solution with a ratio 1 : 1 to keep wt% constant.

### 2.3. Electrophoretic deposition of nanocomposite films

Both Mo_6_ and Nb_6_-based clusters were deposited on indium tin oxide-coated glass (ITO glass) slides acting as anode (A) or cathode (C) electrodes (Geomatec Co., Ltd, Tokyo, Japan; 6.15–7.27 Ω sq^−1^) with a surface area of 1.0 × 2.5 cm^2^. The electric field was generated from a source meter (Keithley Model 2400, Ohio, USA). ITO-coated glass substrates were contacted with electrode bar by using a conductive aluminium double-sided tape. Hybrid films prepared from PMMA and the KNC and KNB clusters dissolved in acetone/water and CMIF cluster dissolved in acetone on the anode side were named A-NC@PMMA, A-NB@PMMA and A-MIF@PMMA respectively. Optimum deposition conditions were found for an applied voltage of 25 V for 1 min and 3 V for 1 min for synthesizing Nb_6_- and Mo_6_-based nanocomposite films respectively.

### 2.4. Characterization

The zeta potential and electric conductivity of MC suspensions were measured by using a zeta-potential analyser (Malvern Instrument, Ltd, Zetasizer Nano Z, Malvern, UK) (electronic supplementary material, table S1). The surface morphology and the elemental composition of the films were analysed by field-emission scanning electron microscopy (FE-SEM, S4800, Hitachi High-Technologies Corp.) at 10 kV coupled with an energy-dispersive X-ray (EDX) analysis device. High-resolution observation of the nanocomposite films was performed by using a transmission electronic microscope (TEM) (JEOL JEM 2100F) also equipped with a mapping EDX analysis and scanning (STEM) devices. The UV-NIR absorption was performed with a V570, Jasco Corp. spectrometer in the wavelength range from 220 to 2000 nm at a scan rate of 400 nm s^−1^. The excitation and PL emission spectra of the CMIF and nanocomposite films were measured by using a high-performance fluorescence spectrometer (FP8500, Jasco Corp.) connected to a xenon lamp at a scan rate of 500 nm s^−1^. The internal quantum efficiency was measured by an absolute PL quantum yield spectrometer C11347 (Hamamatsu Corp.) with the range of the excitation wavelength from 250 nm to 800 nm.

## 3. Results

### 3.1. Characterization of metal atom clusters suspensions

**Figure 1.**
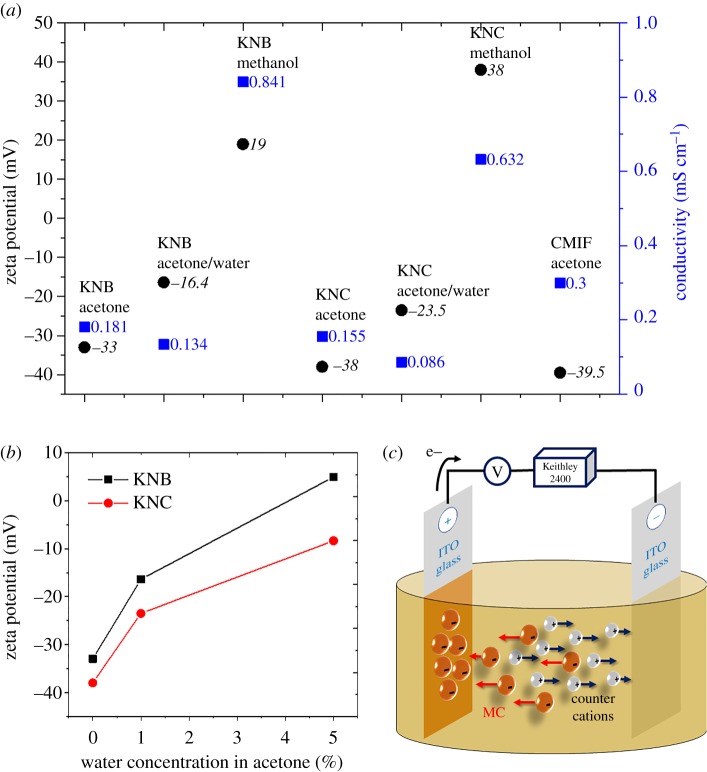
(*a*) Zeta potential (black circles) and conductivity (blue squares) of the MC suspension without polymer in various solvents. (*b*) Evolution of the zeta potential of KNB and KNC clusters in function of the solvent composition; and (*c*) schematic depiction of the anodic EPD process performed with negatively charged clusters.

The electric conductivity of both Nb_6_- and Mo_6_-based clusters suspensions, reported on figure 1*a*, was found to be compatible with the EPD process, in agreement with previous results reported for $Cs_{2}[Mo_{6}Br_{8}^{i}Br_{6}^{a}]$ suspensions [58]. The zeta potential of suspensions depends on the nature of the dispersing medium. Overall, Nb_6_- and Mo_6_-based clusters in acetone exhibited negatively charged surfaces, in good agreement with the chemical formulation of [Nb_6_L^i^_8_L^a^_6_]^n−^ clusters (with *n* = 2, 3 or 4; L = Cl or Br) and [Mo_6_I_8_(OCOC_2_F_5_)_6_]^2−^ clusters **(**figure 1*a***)**. Interestingly, the negative zeta potential of Nb_6_-based clusters increased with the concentration of water in the suspension while it became positive when methanol was used as solvent. This behaviour originates from the exchange of their halogen apical ligands with water, OH^−^ or methanol molecules. Indeed these molecules induce an inversion of the cluster charge (negative to positive) as previously reported for Ta_6_-based clusters in water solution [33]. The kinetic of this exchange process seems to be faster for KNB than for KNC clusters when adding similar amounts of water (figure 1*b*). As a result, both Nb_6_-based octahedral cluster units can exchange their apical ligand with water, OH or solvent molecules, reaching a stable hydrate state in solution in agreement with the literature [66–68]. However, in our study, water also acts as a supporting agent for solubilizing Nb_6_-based clusters in organic solutions. The typical EPD process set-up used for this study, depicted on figure 1*c*, was adapted for depositing negatively charged clusters on the anode (positive electrode).

**Figure 2.**
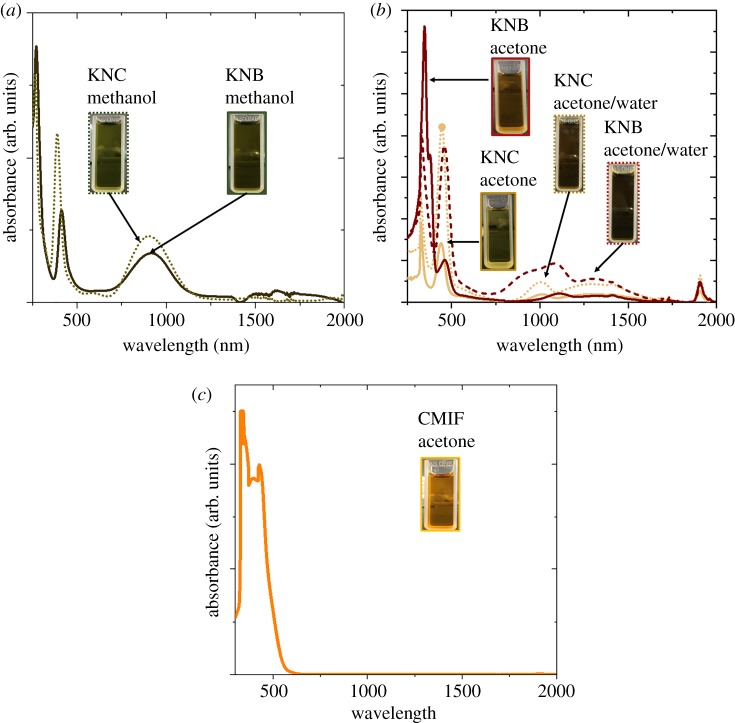
UV–Vis-NIR absorption spectra of KNB and KNC clusters in (*a*) methanol and (*b*) acetone and acetone/water. (*c*) UV–Vis-NIR absorption spectrum of CMIF clusters in acetone.

Figure 2 shows the absorbance of the MC solutions depending on the dispersing media. Suspensions containing Nb_6_-based clusters exhibited a green colour in methanol, owing to their weak absorbance at 350 nm and preferable absorption peak centred at 450 nm in the visible range accompanying an absorption in the NIR region between 750 and 1150 nm (figure 2*a*). Such spectral envelope signals revealed that the structure of Nb_6_-based clusters in methanol remained in a non-oxidized state ${\{Nb_{6}L_{12}^{i}\}}^{2+}$ cluster core (L = Br, Cl). However, the colour of KNB and KNC turned to orange–yellow in acetone suggesting that oxidation of metal clusters occurred (i.e. ${\left\{Nb_{6}L_{12}^{i}\right\}}^{n+}$ cluster core (L = Br, Cl; *n* = 3 or 4)) (figure 2*b*). These units are indeed known for reversible oxidation, causing drastic modulation of their optical properties in the UV–Vis-NIR range yielding yellow-brownish solutions [69–71]. The absorption bands of both clusters followed similar changes in acetone, the three strong transitions at 350 nm, 450 nm and in NIR sharply decreased and new bands appeared in the NIR. Exchanging the apical ligands of MC by water or methanol molecules should lead to new cluster units with chemical formulations such as ${\left[Nb_{6}Cl_{12}^{i}Cl_{6-x}^{a}{\left(L\right)}_{x}^{a}\right]}^{n-4+x}$ and ${\left[Nb_{6}Br_{12}^{i}Br_{6-x}^{a}{\left(L\right)}_{x}^{a}\right]}^{n-4+x}$ (*n* = 0, 1, 2 ; 0 < x < 6; L = H_2_O and/or CH_3_OH) [69,72].

It was also observed that adding a small amount of water (0.01–0.05 ml per 1 ml of acetone) to niobium-based clusters dispersion in acetone leads to the emergence of a second absorption peak around 450 nm, which could explain the brown colour of the suspension. Both the dispersion of MC in acetone and the mitigation of their oxidation were improved in the presence of water (figure 1*b***)**. However, increasing too much the water concentration during the EPD process would lead to solvent hydrolysis along with apical ligand exchange. In the case of the CMIF cluster suspensions in acetone (figure 2*c*), a strong absorption below 500 nm was found, in accordance with the spectrum reported in the literature [60,64]. The oxidation state of the CMIF was more stable than Nb_6_-based clusters as testified by its unchanged colour in acetone, providing advantages for further applications. Mo_6_ cluster thin films were previously assembled by EPD from Cs_2_Mo_6_Br_14_ clusters without supporting polymer [56–58]. However, even with negatively charged clusters, the deposition of KNC, KNB and CMIF films without polymeric binders was not successful in this study within the range of investigated voltages (1–50 V). For instance, when the EPD process was carried on with CMIF, no film deposition was measured but a green colour appeared near the ITO substrate, due to a strong absorption band around 640 nm (electronic supplementary material, figure S1). Further investigations are under progress to understand this result. This unwanted change of optical properties was circumvented by using PMMA as binder to prepare thin films. Upon blending with PMMA as a stabilizer, both the electric conductivity and viscosity of the dispersing medium changed, reducing the mobility of charged particles in the EPD's electric field. After blending, the accurate zeta potential of all suspensions could not be determined. PMMA plays a role as a chemical and dispersion stabilizer, ultimately affecting the mobility of MC.

**Figure 3.**
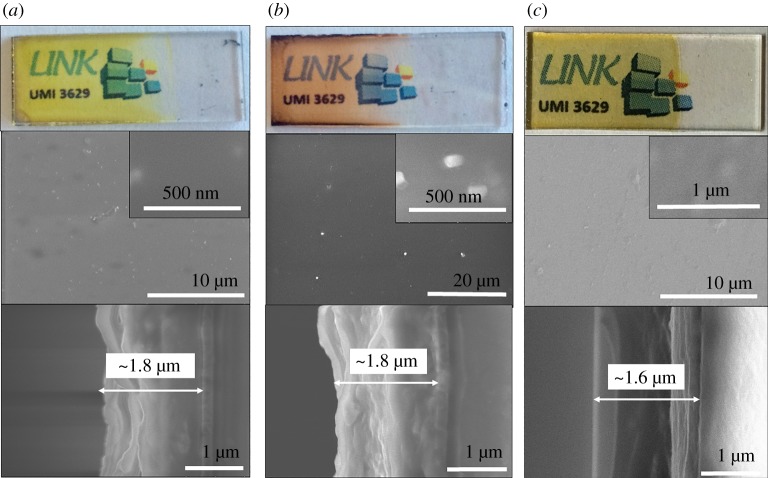
Optical photographs and SEM micrographs of the surface morphology and cross section of hybrid MC films composed of: (*a*) A-NC@PMMA, (*b*) A-NB@PMMA and (*c*) A-MIF@PMMA.

Homogeneous and transparent MC@PMMA films have been successfully prepared by using the EPD process (figure 3). In the case of Nb_6_-based film, it was only possible to obtain films with good quality by using the acetone-water solution. A high transparency level in the visible range was obtained with thin hybrid MC films prepared from A-NC@PMMA, A-NB@ PMMA and A-MIF@PMMA suspensions at different optimal applied voltages. The surface morphology of the films was observed by SEM and revealed defects smaller than 200 nm in Nb_6_-based films, which could originate from the incomplete dispersion of clusters in acetone or some small phase separation due to the addition of water. On the contrary, Mo_6_-based cluster films exhibited a more homogeneous, stable and flat surface without cracks or the roughness typically associated with the use of a PMMA matrix. These improvements are reasonably explained by the better affinity of perfluorinated chains of CMIF with PMMA. The EPD process allowed assembling nanocomposite films thinner than 2 µm, whose composition was tuned with three MC and with a large tolerance to the different applied voltages. These results illustrate the flexibility of the EPD method when applied to the deposition of MC nanocomposite films. Such coatings are easily assembled within short times (60 s) and with a controlled thickness. They can be complementary to other solution coating techniques such as spin and dip coating, drop casting or printing, especially for functionalizing conductive substrates with complex shapes.

**Figure 4.**
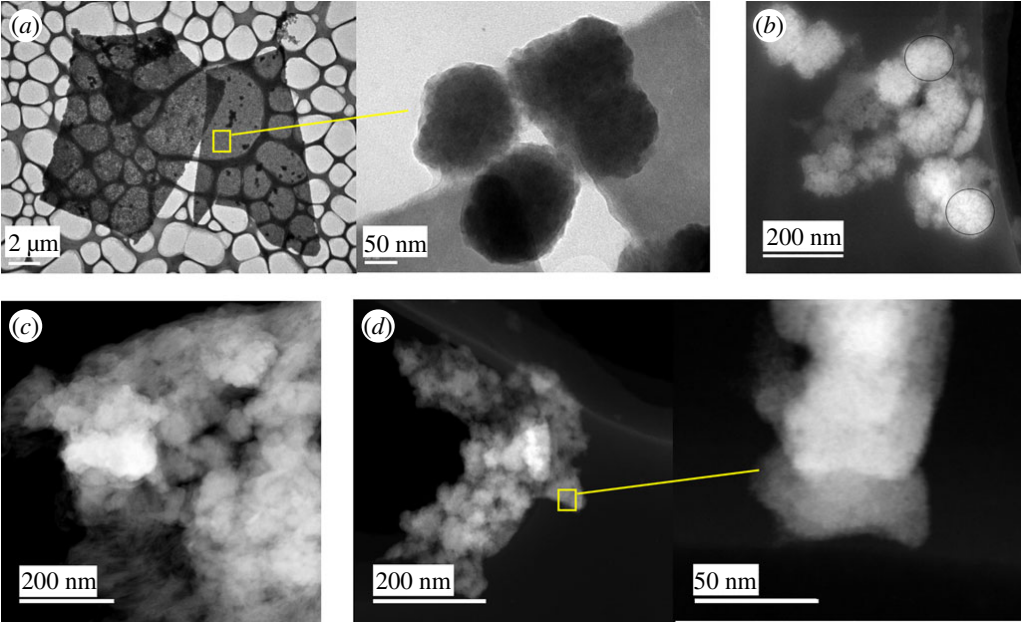
Typical TEM and STEM micrographs of A-NC@PMMA (*a*,*b*), A-NB@PMMA (*c*) and A-MIF@PMMA (*d*) films.

**Figure 5.**
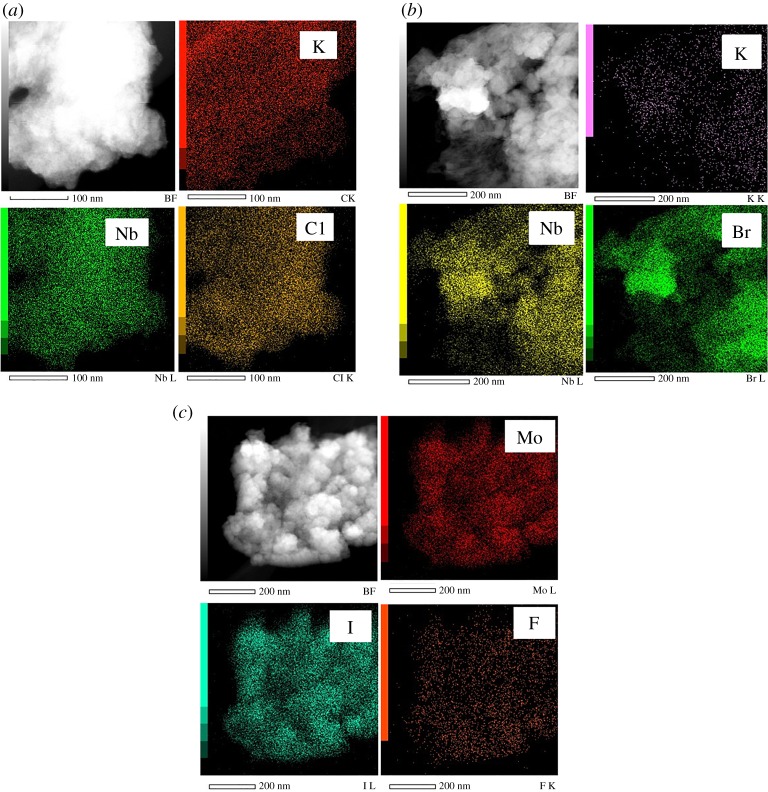
EDX mapping and elemental detection of MC incorporated in: (*a*) A-NC@PMMA, (*b*) A-NB@PMMA and (*c*) A-MIF@PMMA films.

TEM observations of A-NC@PMMA nanocomposite films revealed spherical-shaped aggregates smaller than 200 nm with amorphous intercalation of Nb_6_-based clusters within the PMMA matrix in agreement with SEM experiments. Similarly, STEM micrographs allowed detecting Nb_6_-based clusters as white dots (figure 4*b*,*c*), with a large occupation density in the PMMA matrix, suggesting a relatively good dispersion within the nanocomposite even with the presence of some aggregates. Similar results were obtained with A-MIF@PMMA films. The elemental composition of A-NC@PMMA, A-NB@PMMA and A-MIF@PMMA films was analysed by using EDX mapping (figure 5). Strong signals were obtained for Nb, Mo, Cl, Br, I and F, which are all key components of octahedral MC, as seen in figure 5*a*–*c*. However, this technique is not accurate to give a chemical composition of the nanocomposite due to both its low stability under e-beam irradiation and the high dilution of the considered elements in the PMMA matrix. Counter cations such as Cs^+^ and K^+^ were detected with a weak signal in the films. This result is surprising because, during the EPD process, negatively charged cluster particles migrate to the anode and counter cations to the cathode. The limited mobility of the counter cations towards the cathode may indicate the good intercalation of metal cluster in the polymer.

**Figure 6.**
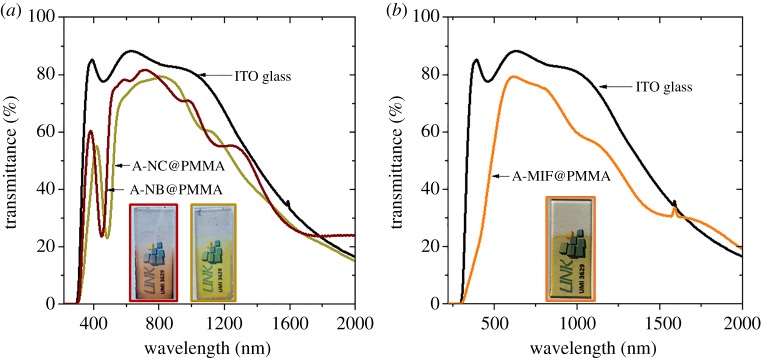
UV–Vis-NIR transmittance spectra of hybrid MC films deposited on ITO-coated glass and composed of (*a*) A-NC@PMMA and A-NB@PMMA, and (*b*) A-MIF@PMMA.

The transmittance of MC@PMMA films deposited on ITO glass was investigated (figure 6). Within the visible range, both A-NC@PMMA and A-NB@PMMA films exhibited strong absorption peaks at 450 and 490 nm, respectively (figure 6*a*). In the case of A-MIF@PMMA films, a strong absorption was observed below 500 nm (figure 6*b*). The UV–Vis-NIR absorption spectra of the MC@PMMA films cumulated the absorption bands coming from MC compounds and from the ITO layer of the glass substrate localized in the NIR region. Ripples were also visible on the spectra, signalling an interference phenomenon typical for homogeneous films [53].

**Figure 7.**
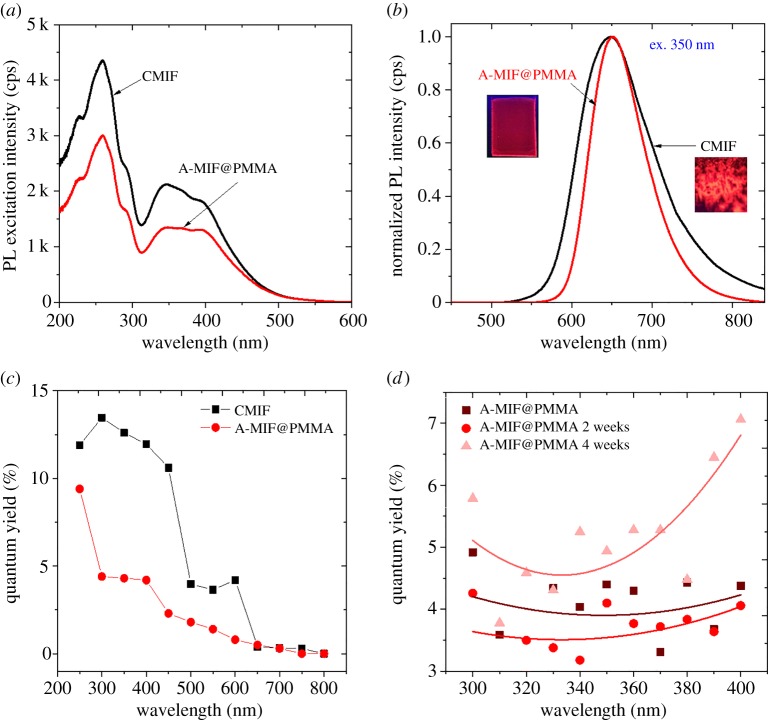
(*a*) Photoluminescence excitation and (*b*) emission spectra (*λ*_ex_ = 350 nm) (the photographs are taken under 365 nm UV lamp). (*c*) Internal quantum yield of CMIF clusters and A-MIF@PMMA films and (*d*) evolution of the quantum yield of the A-MIF@PMMA film upon exposure to 70% RH at 50°C for two and four weeks.

The PL excitation and emission spectra of A-MIF@PMMA films and CMIF clusters powder were studied (figure 7*a*,*b*). Both Mo_6_ clusters-based materials exhibited a similar excitation and emission spectra before and after the EPD (figure 7*a*,*b*). This result is in good agreement with previous results which have shown that the PMMA matrix has no or very low impact (less than 240 cm^−1^ shift) on the maximum of the PL emission peak of Mo_6_ clusters [42,43].

The quantum yield of CMIF clusters, either as a pure powder or as A-MIF@PMMA films, was investigated (figure 7*c*). As expected, in both cases, the higher the excitation wavelength, the lower the emission efficiency. This trend was, however, more accentuated for the CMIF cluster. The decrease of the quantum yield of Mo_6_ clusters after incorporation in PMMA matrix was already reported [42,43]. As the quantum yield depends on the ratio of the emission and the absorption energy, we propose that the additional energy absorption in A-MIF@PMMA films originates from the π orbital linking of the PMMA chains. Moreover, Amela-Cortes *et al*. [42] found that the quantum yield drastically decreases with the increase of the cluster content (CMIF > 10 wt%). Even, if estimating the final Mo_6_/PMMA ratio in the film is difficult, the lower quantum yield observed could signal that the EPD process allowed reaching a high content of Mo_6_ cluster in the film. Finally, the reduced emission intensity of the cluster film could originate from the MC instability: some apical (COOC_2_F_5_) groups of the CMIF octahedral clusters could be replaced by water molecules (impurities in acetone solvent) during the cluster dispersion and the EPD process that is in good agreement with the small shift observed for the cluster emission band maximum after integration. The cluster's behaviour within the polymer matrix will be confirmed in a future study. The stability of the PL emission was investigated by keeping the films in specific conditions of temperature and relative humidity (RH). A-MIF@PMMA films were dried for one week at ambient condition and then kept in a sealed chamber for two to four weeks at a temperature of 50°C and an RH of 70%. The quantum yield of MIF@PMMA films was recorded between 300 and 400 nm before and after being exposed to these conditions (figure 7*d*). The quantum yield remained almost constant after two and four weeks of exposure, indicating the crucial role of PMMA in stabilizing the clusters by preventing the exchange of the apical ligand of Mo_6_ octahedral cluster unit by water molecules, even at high RH (70%). This result supports the hypothesis of an enhanced stability of the clusters when hybrid MC@PMMA nanocomposite film is prepared.

**Figure 8.**
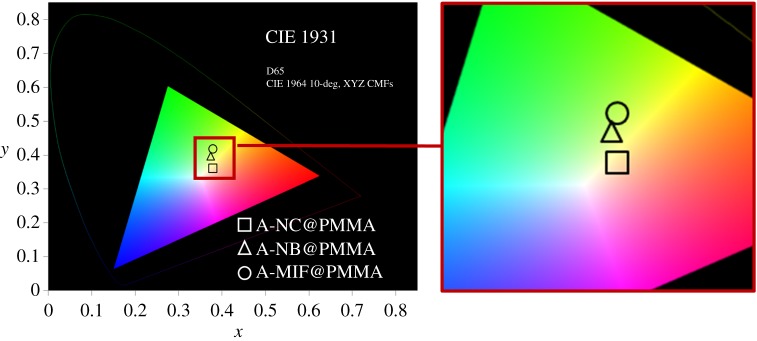
CIE chromaticity coordinates of the MC@PMMA films prepared by the EPD process.

In order to evaluate the efficiency of the cluster coating as energy-saving materials, different figure of merit (FOM) values (*T*_vis_, *T*_sol_ and *T*_vis_/*T*_sol_) (table 1; electronic supplementary material) and CIE colour coordinates (*x, y*) (figure 8) of the Nb_6_ or Mo_6_@PMMA films were calculated by using a previously developed equation [33]. *T*_sol_ is the integrated spectral transmittance of a window weighted with the normalized solar energy distribution spectrum, whereas *T*_vis_ was calculated in a similar way, but weighted with the photopic response of the human eye [73–76]. The *x* and *y* values are in agreement with the observed colour of the films. For smart windows technologies, cumulating the highest transparency and efficiency requires to selectively harvest all of the invisible parts of the solar spectrum, including the near-infrared (NIR) and ultraviolet (UV) [77,78]. All the MC@PMMA films reached *T*_vis_ values (table 1) much higher than 50% as requested for windows application [74], which demonstrate the high transparency in the visible for these films. The A-NB@PMMA film exhibits a *T*_vis_/*T*_sol_ ratio of 1.07, lower than ITO-coated glass (1.12), and corresponding to the dark brown colour of the film. Nevertheless, the *T*_vis_/*T*_sol_ ratio for Nb_6_-based cluster film could be improved by controlling the dispersion and the oxidation state of the Nb_6_ clusters. On the contrary, both A-NC@PMMA and A-MIF@PMMA films obtained improved *T*_vis_/*T*_sol_ ratios, reaching 1.19 and 1.24, respectively, with an improvement of *T*_sol_ of about 25% comparing pure ITO-coated glass. The high *T*_vis_/*T*_sol_ ratio of these films indicates the crucial attenuation of the UV-NIR solar energy and demonstrates the efficiency of MC@PMMA films as an environment-friendly material.

**Table 1.** FOM values and colour coordinates of MC@PMMA films. ITO@glass is used as reference [31].

name	*x*	*y*	*T*_vis_	*T*_sol_	*T*_vis_*/T*_sol_
A-NC@PMMA	0.378	0.419	74.1	62.2	1.19
A-NB@PMMA	0.379	0.362	61.7	57.7	1.07
A-MIF@PMMA	0.374	0.397	71.1	57.5	1.24
ITO@Glass	0.318	0.340	87.6	78.0	1.12

## 4. Conclusion

Different nanocomposites with red-NIR luminescent and/or UV/NIR blocking properties based on Mo_6_ and Nb_6_ clusters and PMMA were deposited on ITO-coated glass by using the EPD process. MC compounds are integrated without any chemical modifications in the PMMA matrix up to 29 wt% for Mo_6_ and 6wt% for Nb_6_ clusters. The crucial role of PMMA as a polymeric binder for improving the processability of EPD was demonstrated, allowing stabilizing the optical properties of the films. All MC@PMMA nanocomposite films were homogeneous at the microstructural level with a good cluster dispersion within the polymer matrix, ultimately allowing reaching a high transparency in the visible range. The Mo_6_@PMMA films exhibited a strong and durable red emission even after exposure to quite elevated temperature (50°C) and RH (70%) for four weeks. The cluster's oxidation state and the control of the exchanged apical ligands seems to be the most important parameter for improving the UV-NIR absorption of Nb_6_@PMMA films. Mass spectrometry and density functional theory calculations are in progress to fully understand this behaviour (not presented here). Nanocomposite films based on A-NC@PMMA and A-MIF@PMMA exhibited promising properties for attenuating the UV-NIR solar energy. Owing to these UV-NIR blocking properties, nanocomposite films based on Nb_6_ and Mo_6_ cluster and polymeric binders will be a promising material for optical window application in the future.

## Supplementary Material

Synthesis of metal cluster

## Data accessibility

The datasets supporting this article have been uploaded as part of the electronic supplementary material.
