# Supplementary material for: Transparent functional nanocomposite films based on octahedral metal clusters: synthesis by electrophoretic deposition process and characterization
Source: R Soc Open Sci. 2019 Mar 6;6(3):181647. doi: 10.1098/rsos.181647 (PMC6458394; doi:10.1098/rsos.181647)
Supplement: Synthesis of metal cluster [file rsos181647supp1.docx]

**SUPPORTING INFORMATION**

Transparent functional nanocomposite films based on octahedral metal clusters: synthesis by electrophoretic deposition process and characterization

**Ngan Thi Kim Nguyen^1,2^, Marion Dubernet^1,2^, Yoshio Matsui^1^, Maxence Wilmet^2,3^, Naoto Shirahata^4^, Gaulthier Rydzek^4,5^, Noée Dumait^3^, Maria Amela-Cortes^3^, Adèle Renaud^3^, Stéphane Cordier^3^, Yann Molard^3^, Fabien Grasset^1,2^ and Tetsuo Uchikoshi^1,2^**

*^1)^ Research Center for Functional Materials, National Institute for Materials Science (NIMS), 1-2-1 Sengen, Tsukuba, Ibaraki 305-0047, Japan*

*^2)^ Laboratory for Innovative Key Materials and Structures (LINK), UMI 3629 CNRS-Saint-Gobain-NIMS, National Institute for Materials Science, 1-1 Namiki, Tsukuba, Ibaraki 305-0044, Japan*

^3)^ Univ Rennes, CNRS, ISCR – UMR 6226, F-35000 Rennes, France

^4)^ Research Center for Materials Nanoarchitectonics (MANA), National Institute for Materials Science (NIMS), 1-1 Namiki, Tsukuba 305-0044, Japan

*^5)^ Centre for Research on Adaptive Nanostructures and Nanodevices (CRANN) and Advanced Materials Bio-Engineering Research Centre (AMBER), School of Chemistry, Trinity College Dublin, Dublin, Ireland*

*Experimental*

*Preparation of Cs_2_Mo_6_I_8_(C_2_F_5_COO)_6_ Metal Clusters [1-2]*

The Cs_2_Mo_6_I_8_(C_2_F_5_COO)_6_ cluster compound was prepared from Cs_2_Mo_6_I_14_ and AgOCOC_2_F_5_.

Cs_2_Mo_6_I_14_: First, MoI_2_ starting compound was synthesized by heating a stoichiometric mixture of Mo (Plansee 99.8 %) and I_2_ (Alfa Aesar 99.8 %) at 700 °C for 4 days in a silica tube (noted SiO_2_ in the sketch) sealed under vacuum. Afterwards, Cs_2_Mo_6_I_14_ was prepared using CsI (Alfa Aesar 99.9 %) and MoI_2_. The mixture (0.5 g) was ground, formed as a pellet and placed into silica tube (o.d. 9 mm, i.d. 7 mm, length 70 mm). Once sealed under vacuum, the tube was heated for three days at 700 °C. The X-ray powder pattern of the final product did not evidence the presence of any impurity. Red thin plate-shaped crystals of Cs_2_Mo_6_I_14_ were obtained after a 100 °C/day cooling rate of temperature.

Cs_2_Mo_6_I_8_(C_2_F_5_COO)_6_: To a solution of Cs_2_Mo_6_I_14_ (1.5 g, 0.52 mmol) in 20 mL of acetone, was added a solution of silver pentafluoropropionate (0.935 g, 3.42 mmol) in 10 mL of acetone under argon and in the dark. The mixture was stirred for 48 h in the dark and then was filtered through a Celite® pad. The red solution was then evaporated to yield a red-orange powder. Yield = 97%. ^19^F-NMR (acetone-d_6_): δ (ppm) = −83 (3F), −120 (2F). EDAX: Cs 2, Mo 8, I 11, F 77, no Ag.

Single-crystal X-ray diffraction data of Cs_2_Mo_6_(C_2_F_5_COO)_6_ and Cs_2_Mo_6_I_14_ were collected at room temperature on a Bruker AXS APEX-II diffractometer or a Nonius KappaCCD X-ray area-detector diffractometer with Mo Kα radiation (λ = 0.71073A) respectively.

**

Representation according to single crystal data X-ray diffraction measurements of Cs_2_Mo_6_I_8_(C_2_F_5_OCO)_6_.

*Preparation of K_4_Nb_6_X_18_ metal clusters [3]*

They were obtained by reduction of the pentahalogenated precursor by metallic niobium in an alkaline medium at high temperature under neutral atmosphere. Thus, NbBr_5_, Nb and KBr were mixed together in a glovebox and put into a silica sealed under vacuum tube. K_4_Nb_6_X_18_ phase was obtained after sintering at 600°C during 24 hours in a rocking furnace. Niobium and recrystallized KBr impurities were then removed by dissolution of synthesized powder (1.5 g) in absolute ethanol (55 mL) and filtration of the solution.

Figure S1. UV-Vis absorption spectrum of CMIF clusters in acetone during EPD.


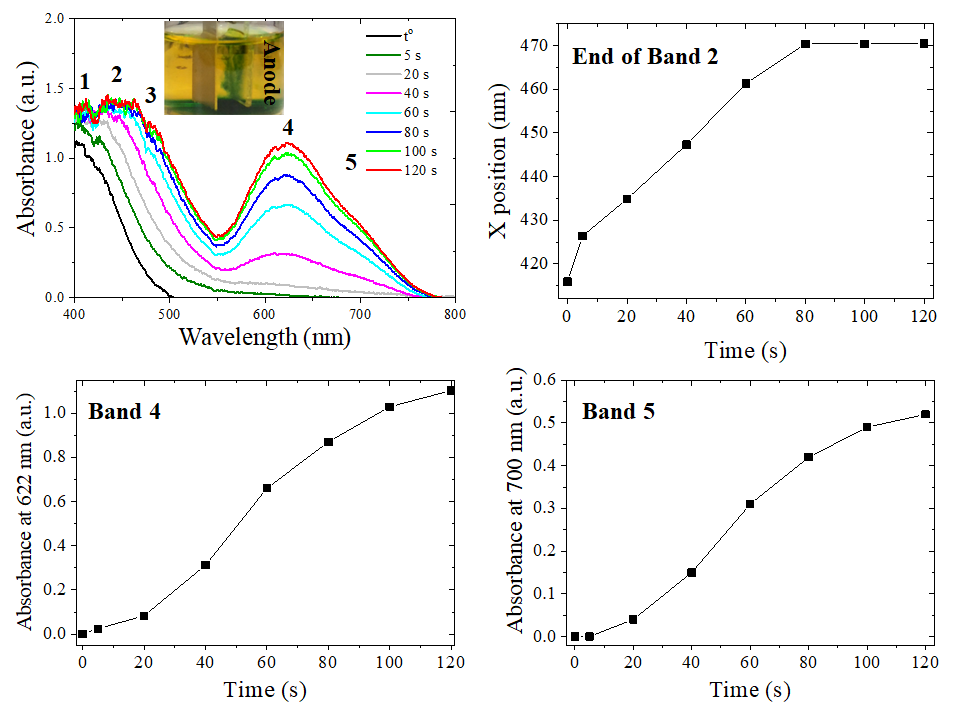


1. M. Amela-Cortes, Y. Molard, S. Paofai, A. Desert, J.-L. Duvail, N.G. Naumov, S. Cordier, *Dalton Trans*., 2016, **45**, 237
2. K. Kirakci, S. Cordier, C. Perrin, *Z. Anorg. Allg. Chem.*, 2005, **631**, 411
3. F. W. Koknat, J. A. Parson, A. Vongvusharintra, Inorg. Chem.1974, 13, 1699
